# A macroscopic clock model to solve the paradox of Schrödinger’s cat

**DOI:** 10.1038/s41598-024-53833-3

**Published:** 2024-02-13

**Authors:** D. Sokolovski, A. Uranga, E. Akhmatskaya

**Affiliations:** 1 Departmento de Química-Física, Universidad del País Vasco, UPV/EHU, 48940 Leioa, Spain; 2 Basque Center for Applied Mathematics (BCAM), Alameda de Mazarredo 14, 48009 Bilbao, Spain; 3 IKERBASQUE, Basque Foundation for Science, Plaza Euskadi 5, 48009 Bilbao, Spain; 4 EHU Quantum Center, Universidad del País Vasco, UPV/EHU, 48940 Leioa, Spain

**Keywords:** Physics, Quantum physics, Quantum mechanics

## Abstract

We propose detecting the moment an atom emits a photon by means of a nearly classical macroscopic clock and discuss its viability. It is shown that what happens in such a measurement depends on the relation between the clock’s accuracy and the width of the energy range available to the photon. Implications of the analysis for the long standing Schrödinger’s cat problem are reported.

## Introduction

In non-relativistic quantum mechanics, time is a mere parameter, quite distinct from the dynamical variables such as positions and momenta, conveniently represented by Hermitian operators. This often complicates the queries, easily answered in the classical context (a good overview has been given in Refs.^1^ and^2^). When does a quantum particle arrive at a given location (see Egusquiza, Muga and Baute in^1^ and Galapon in^2^)? How much time does a tunnelling particle spend in the barrier (see, e.g.,^3,4^)? How long does a quantum jump take (see Schulman, in^5^ and Refs. therein)? These questions continue to cause controversy, and here we add one more to the list.

If an atom, initially in an excited state, emits a photon and is later found in its ground state, when exactly did the transition take place? If the decay sets off a chain of events leading to the death of a cat^6^, how long ago did the cat die? This is another general problem in elementary quantum mechanics, and below we will address it, using the simplest model available. Here we take the view found, e.g., in^7^. For any observed sequence of events quantum mechanics provides a probability amplitude, essentially a matrix element of an unitary evolution operator between the states representing the observed conditions. Its absolute square yields the desired likelihood of seeing an outcome or outcomes. At the end of the experiment an observer has access to the atom’s condition, plus the record of the moment of decay, encoded into the clock’s (the cat’s) condition. Whether quantum mechanics can be expected to do more is, indeed, an open question beyond the scope of our paper.

### A meaningful question?

Does it make sense to talk about the moment the atom decayed? Not always. Decay of a metastable state is often described by a model^8,9^ where a discrete state $|e{\rangle }$, corresponding to an excited “atom” with energy $E_{e}$, is connected to “reservoir” states $\{|E_r{\rangle }\}$, representing an “atom” in its ground state, $E_g=0$, plus an emitted “photon” with energy $E_r$. The corresponding Hamiltonian takes the form,

1 $$\begin{aligned} \hat{H}=\hat{H}_0 +\hat{V},\quad \hat{H}_0=|{e}{\rangle }E_{e}{\langle }{e}| + \sum _r |E_r{\rangle }E_r{\langle }E_r|,\nonumber \\ \hat{V}= \sum _r \Omega (E_r)\left( |E_r{\rangle }{\langle }{e}| + |{e}{\rangle }{\langle }E_r|\right) , \quad \quad \quad \end{aligned}$$

where $\Omega (E_r)$ is the matrix element responsible for the transitions between the system’s discrete and continuum states, i.e., for the decay of the excited atom. In the continuum limit, whenever the final probabilities are added up, one can replace the sum $\sum _r$ by an integral $\int \rho (E_r) dE_r$, where $\rho (E_r)$ is the density of the reservoir states^8^.

After preparing an atom in its excited state, and waiting for *t* seconds, one can find a photon with an energy $E_r$. Expanding the transition amplitude ${\langle }E_r|\exp (-i\hat{H}t)|{e}{\rangle }$ in powers of $\hat{V}$ reveals a variety of scenarios where the photon, emitted for the first time at $\tau _{\textrm{first}}$ is re-absorbed and re-emitted until settling down into its final state $|E_r{\rangle }$ at some $\tau _{\textrm{last}}$. Thus, the emission process may not occur via a single transition to the ground state, but can have a finite duration $\tau _{\textrm{last}}-\tau _{\textrm{first}}$. Measuring even “the first passage time” $\tau _{\textrm{first}}$ presents considerable difficulties^10^, and we do not know if $\tau _{\textrm{last}}-\tau _{\textrm{first}}$ can be measured at all.

A helpful exception is the first order transition in the weak coupling limit, which does indeed occur via a single jump,

2 $$\begin{aligned} {\langle }E_r|\exp (-i\hat{H}t)|{e}{\rangle }=-i\Omega (E_r) \int _0^t d\tau \exp [-iE_r(t-\tau )]\exp (-iE_{e}\tau )+ \mathscr {O}(V^3), \end{aligned}$$

yet the jump’s precise moment remains indeterminate due to the Uncertainty Principle^7^. One way to pinpoint the time of transition is to subject the atom to frequent observations every $\delta t =t/K$, $K>>1$. This, however, is known to lead to the Zeno effect which quenches the transition, whose rate changes from the one given by the Fermi’s golden rule^11^ for an unobserved atom, $\Gamma _{Fermi} =2\pi |\Omega (E_{e})|^2{\rho }(E_{e})$ to $\Gamma _{\delta t} \approx \delta t\times \int _{-\infty }^\infty dE {\rho }(E)|\Omega (E)|^2$, which vanishes as $\delta t \rightarrow 0$ (see, e.g.,^5^).

Yet, there is a case where the transition proceeds via a single jump, and the Zeno effect does not occur. We will discuss it next.

## Results and discussion

### The wide band (Markovian) case

In the Markovian (wide band) approximation^8^, both $\Omega (E_r)$ and ${\rho }(E_r)$, are taken to constant, very small and very big respectively, i.e. $\Omega \rightarrow 0$, ${\rho }\rightarrow \infty$, in such a manner that a product ${\rho }\Omega ^2$ remains finite,

3 $$\begin{aligned} 2\pi {\rho }\Omega ^2\equiv \Gamma < \infty . \end{aligned}$$

The model admits an exact solution for any $\Gamma$, and there is no need to limit oneself to the first order approximation (5). The amplitudes of the four possible processes are given by^8^:

4 $$\begin{aligned}{} & {} {\langle }{e}|\exp (-i\hat{H}t)|{e}{\rangle }= \exp (-iE_{e}t -\Gamma t/2),\end{aligned}$$

5 $$\begin{aligned}{} & {} {\langle }E_r|\exp (-i\hat{H}t)|{e}{\rangle }=-i\Omega \int _0^t dt' \exp [-iE_r(t-t')]\exp (-iE_{e}t'-\Gamma t'/2), \end{aligned}$$

6 $$\begin{aligned}{} & {} {\langle }{e}|\exp (-i\hat{H}t)|E_r{\rangle }=0,\quad \text {since}\quad \Omega \rightarrow 0, \end{aligned}$$

7 $$\begin{aligned}{} & {} {\langle }E_{r'} |\exp (-i\hat{H}t)|E_r{\rangle }=\exp (-iE_rt)\delta _{rr'},\quad \text {since}\quad {\langle }E_{r'}| \hat{H}|E_r{\rangle }=E_r\delta _{rr'}. \end{aligned}$$

By (4), atom’s decay is exponential at all times, and by (5), the energy distribution of the emitted photons is Lorentzian

8 $$\begin{aligned} P(E_r\leftarrow {e}, t\rightarrow \infty )=\frac{\rho \Omega ^2}{(E_r-E_{e})^2+\Gamma ^2/4}. \end{aligned}$$

Further helpful to our purpose is the fact that, according to Eqs. (5) and (6), the atom can emit a photon only once, and never re-absorbs it afterwards. The moment of transition can, therefore, be defined at least in terms of the virtual scenarios available to the system. With the purely exponential decay in Eq. (4) frequent checks of the atom’s state do not affect the decay rate, which stays the same with or without such checks [hence, the adjective *Markovian*, $P^{\text {decay}}_M( t) = 1- \exp (-\Gamma t)=1- [\exp (-\Gamma t/K)]^K= P^{\text {decay}}_{\delta t}(t)$]. Even so, destruction of coherence between the moments of emission in Eq. (5) must change something akin to the interference pattern in a double slit experiment. Below we will show that it is the energy spectrum of the emitted photons (8) that is affected by the measurement’s accuracy.

### A quantum hourglass and its macroscopic limit

**Figure 1:**
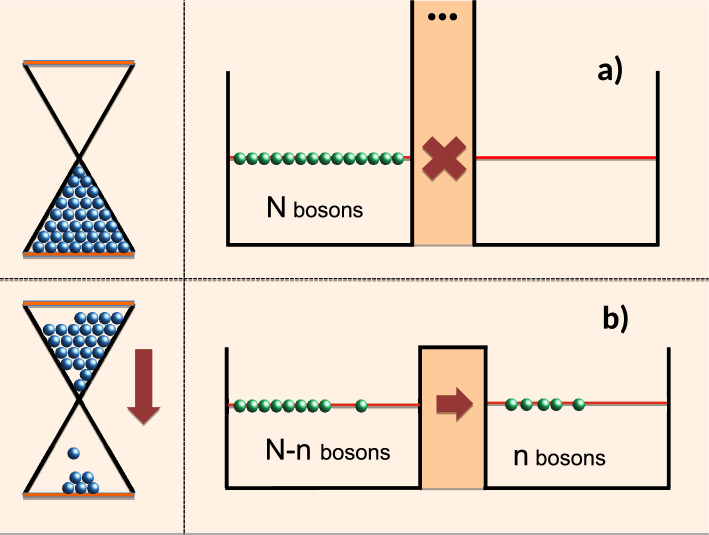
A classical hourglass (left), and its quantum version (right). (**a**) With the barrier closed (the clock is switched off) the bosons remain in the left well. (**b**) If the barrier is down (the clock is switched on), the number of bosons escaping into the right well allows one to estimate the elapsed time.

Suppose Alice the experimenter, does not wish to subject the system to frequent checks, and prefers instead to have, at the end of the experiment, a single record of the moment the atom decayed. For this purpose, she might consider a clock which stops at the moment the atom leaves its excited state. The clock could be an hourglass, in which case the number of the sand grains escaped, would tell Alice the time of the event. A quantum analogue of an hourglass is not difficult to find. Alice could use an array of identically polarised distinguishable spins precessing in a magnetic field, and estimate the elapsed time by counting the spins which have been flipped. Alternatively, Alice can employ a large number of non-interacting bosonic atoms, $N>>1$, initially in the left well of a symmetric double well potential (see Fig. 1).

The clock Hamiltonian given by

9 $$\begin{aligned} \hat{H}^{\text {clock}}=\omega \bigg [{\hat{a}}^+_R {\hat{a}}_L+{\hat{a}}^+_L {\hat{a}}_R \bigg ], \end{aligned}$$

where ${\hat{a}}^+_{R(L)}$ creates a boson in the right (R), or left (L) well, and $\omega$ is the hopping matrix element and the amplitude of finding *n* bosons in the right well is easily found to be

10 $$\begin{aligned} A^{\text {clock}}_{\text {Bose}} (n\leftarrow 0,t)= (-i)^n\sqrt{C^N_n p^n{(t)}(1-p{(t)})^{N-n}}, \quad p(t) \equiv \sin ^2(\omega t), \end{aligned}$$

where $C^N_n=\frac{N!}{n!(N-n)!}$ is the binomial coefficient.

Alice can choose $\omega t<<1$, so that the Rabi period of a single boson is very large, and have a practically irreversible flow of bosons from left to right. She can also assure, by making *N* very large, that the mean number of atoms in the right well is also large (except perhaps at very short times), $\overline{n}(t)\equiv p(t) N>>1$. Under these conditions, binomial distribution under the root sign in (10) can be approximated by a normal distribution^12^, and after some algebra [see the “Methods” section (Derivation of Eq. (11))] we have

11 $$\begin{aligned} A^{\text {clock}}_{\text {Bose}}(n\leftarrow 0,t)\approx \frac{(-i)^n}{[2\pi n]^{1/4}}\exp \left[ -\frac{(t_n-t)^2}{\Delta t^2}\right] ,\quad t_n \equiv \omega ^{-1}\sqrt{n/ N}, \quad \Delta t =\omega ^{-1}N^{-1/2}. \end{aligned}$$

**Figure 2:**
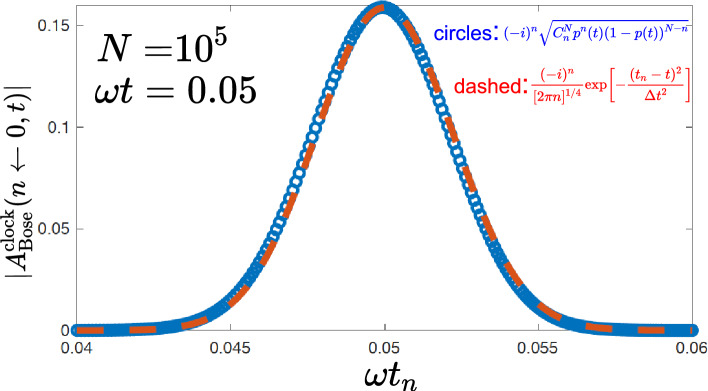
Comparison between Eqs. (10) and (11). Out of $N=10^5$ bosons, 250 are in the right well..

Alice can now count the atoms in the right well and use $t_n$ in Eq. (11) as an estimate for the elapsed time. Equation (11) shows that her estimate is likely to be within an error margin $\Delta t$ of the true value *t*. A good clock is the one which has a small relative error. If $\omega t$ is kept constant while $N\rightarrow \infty$, the error tends to zero, since $\Delta t/t_n =1/\sqrt{n} \approx 1/(\omega t \sqrt{N}) \sim 1/\sqrt{N}$, and with many bosons Alice has a good clock (see Fig. 2).

We make a further remark. As $N\rightarrow \infty$, a large system of independent particles begins to develop certain classical properties^13,14^ [see also the “Methods” section (Derivation of Eq. (11))]. For example, denoting one-partial states in the wells as $|L{\rangle }$ and $|R{\rangle }$, and preparing the bosons in a state $|\Phi ^{\text {clock}}_{\text {Bose}}(0){\rangle }=\prod _{i=1}^N|L{\rangle }_i$, one later finds them in $|\Phi ^{\text {clock}}_{\text {Bose}}(t){\rangle }=\prod _{i=1}^N[u_{LL}(t)|L{\rangle }_i+u_{RL}(t)|R{\rangle }_i]$, where $u_{LL}(t)=\cos (\omega t)$ and $u_{RL}(t)=-i\sin (\omega t)$ are the matrix elements of the one-particle evolution operator. The evolved state $|\Phi ^{\text {clock}}_{\text {Bose}}(t){\rangle }$ is not an eigenstate of an operator $\hat{n} =\sum _{i=1}^N |R{\rangle }_i {\langle }R|_i=\hat{a}^+_R\hat{a}_R$, which gives the number of bosons in the right well. However, expanding it in the eigenstates of $\hat{n}$, $\hat{n} |n{\rangle }=n|n{\rangle }$, $n=0,1 \ldots N$, one finds^14^ the coefficients localised in a range $\sim \sqrt{N}$ around a mean value $\overline{n}(t) \equiv {\langle }\Phi ^{\text {clock}}_{\text {Bose}}(t)|\hat{n}|\Phi ^{\text {clock}}_{\text {Bose}}(t){\rangle }= N\sin ^{2}(\omega t)\approx N\omega ^2t^2{\propto N}$,

12 $$\begin{aligned} {\langle }n|\Phi ^{\text {clock}}_{\text {Bose}}(t){\rangle }\approx \frac{(-i)^n}{\left[ 2\pi \overline{n}(t)\right] ^{1/4}}\exp \left[ -\frac{\left( n-\overline{n}(t)\right) ^2}{2\overline{n}(t)}\right] . \end{aligned}$$

A similar localisation would occur if $|\Phi (t){\rangle }$ expanded in any basis, and this has important consequences. Firstly, one can accurately measure $\hat{n}$ (or any other operator^14^) and obtain a result close to its mean value ($\sim N$) with an error margin $\sim \sqrt{N}$. This is a good measurement, since its relative error tends to zero. Secondly, one can measure it inaccurately, e.g., by using a von Neumann pointer prepared in a Gaussian state of a width $\sim N^{1/2+\varepsilon }$, where $0<\varepsilon < 1/2$^14^. This is still a good measurement since $\sim N^{1/2+\varepsilon }/N\rightarrow 0$, but also one which in the limit $N\rightarrow \infty$ leaves the state (12) almost intact, since $N^{1/2+\varepsilon }/\sqrt{N}\rightarrow \infty$ [see the “Methods” section (A macroscopic clock) for details]. Alice can keep reading this macroscopic nearly classical clock without affecting its operation, like she would do with a classical wrist watch.

### A clock which first runs and then stops

Next Alice needs to make the clock run until the moment the atom emits a photon. This can be achieved by coupling it to the atom-photon Markovian system (*M*) by means of a Hamiltonian

13 $$\begin{aligned} \hat{H}^{\text {a+ph+clock}} = \hat{H}_{M} + \hat{\pi }_e \hat{H}^{\text {clock}}, \quad \hat{\pi }_e \equiv |e{\rangle }{\langle }e|, \end{aligned}$$

where $\hat{\pi }_e$ projects onto the atom’s excited state (for possible realisation of coupling a Bose–Einstein condensate to quantum dots see, e.g.^15–17^). The corresponding Schrödinger equation is easily solved, and the amplitude for the composite $\{\text {(a)tom+(ph)oton + clock}\}$, starting with the right well empty, to end with *n* bosons there, is found to be [see the “Methods” section (Coupling the clock to a quantum system)]

14 $$\begin{aligned} A^{\text {a+ph+clock}}_{\text {Bose}}(j,n\leftarrow e,0) = \int _0^t A^{\text {clock}}_{\text {Bose}}(n\leftarrow 0,\tau ) A^{\text {a+ph}}(j\leftarrow e,{t|\tau }) d\tau , \nonumber \\ j=e\, \text{ or }\, E_r,\quad n=0, \ldots N, \end{aligned}$$

where $A^{\text {clock}}_{\text {Bose}}(n\leftarrow 0,\tau )$ is given by Eq. (11), and $A^{\text {a+ph}}(j\leftarrow e,{t|\tau })$ is the amplitude for the atom-photon system to reach a final state $|e{\rangle }$ or $|E_r{\rangle }$ after remaining in $|e{\rangle }$ for exactly $\tau$ seconds,

15 $$\begin{aligned} A^{\text {a+ph}}(j\leftarrow e,{t|\tau }) ={\langle }j|\hat{U}^{\text {a+ph}}(t|\tau )|e{\rangle },\quad \quad \quad \quad \quad \nonumber \\ \hat{U}^{\text {a+ph}}(t|\tau )\equiv (2\pi )^{-1}\int _{-\infty }^\infty \exp [i\lambda \tau -i(\hat{H}_{M}+\lambda \hat{\pi }_e)t ] d\lambda , \end{aligned}$$

where $\hat{U}^{\text {a+ph}}(t|\tau )$ is the conditional evolution operator. This is clearly the desired result. The clock runs only while the atom remains in the excited state, and the amplitudes are added for all possible durations $\tau$, which may lie between 0 and *t*. The integral in Eq. (15) is evaluated by noting that adding $\lambda \hat{\pi }_e$ to $\hat{H}_{M}$ only shifts the energy of the discrete state $E_e$ by $\lambda$ [see the “Methods” section (Timing the transition in the Markovian case)]. The result ($0\le \tau \le t$)

16 $$\begin{aligned} A^{\text {a+ph}}(e\leftarrow e,t|\tau ) &=\exp (-iE_e t -\Gamma t/2)\delta (\tau -t),\quad \quad \quad \quad \quad \quad \quad \nonumber \\ A^{\text {a+ph}}(E_r\leftarrow e,t|\tau ) &=-i\Omega \exp [-iE_r(t -\tau )] \exp [-i (E_e-i\Gamma /2) \tau ] \end{aligned}$$

confirms what is already known from Eqs. (4) and (5). An atom, still found in the excited state at *t*, has remained in that state all the time. An atom, found in the ground state, has not returned to the excited state after making a single transition at some $\tau$ between $t=0$ and *t*.

Alice the practitioner can now prepare the atom in its excited state, couple it with a “good” clock (11), wait until time *t*, and then measure the energy of the photon (if any), as well as count the bosons in the right well. She can find no photon and *n* bosons, with a probability

17 $$\begin{aligned} P(e,n\leftarrow e,0) =\exp (-\Gamma t)P^{\text {clock}}_{\text {Bose}}(n\leftarrow 0,t), \quad \sum _{n=0}^N P_{\text {Bose}}(n\leftarrow {0},t)=1, \end{aligned}$$

where $P^{\text {clock}}_{\text {Bose}}(n\leftarrow 0,t)=|A^{\text {clock}}_{\text {Bose}}(n\leftarrow 0,t)|^2$ [see Eq. (11)]. She may find *n* bosons, a photon with an energy $E_r$, and conclude that the emission occurred around [see Eq. (11)]

18 $$\begin{aligned} \tau _n =\omega ^{-1}\sqrt{n/N}. \end{aligned}$$

The error of this result is determined by the width of the Gaussian (11) which, restricts the possible values of $\tau$ in Eq. (14). Alice’s relative error is, therefore, $\Delta t /\tau _n \sim 1/\sqrt{n}<<1$, where $\Delta t =\omega ^{-1}N^{-1/2}$ was defined in Eq. (11). The probability of this outcome is given by the absolute square of $A^{\text {a+ph+clock}}_{\text {Bose}}(E_r,n\leftarrow e,0)$ in Eq. (14). Extending in Eq. (14) the limits of integration to $\pm \infty$, and evaluating Gaussian integrals yields

19 $$\begin{aligned} P(E_r,\tau _n\leftarrow e,0)\approx \frac{\pi \Omega ^2}{\omega \sqrt{nN}}\exp (-\Gamma \tau _n)\times \frac{\Delta t}{\sqrt{2\pi }}\exp [-(E_r-E_{e})^2\Delta t^2/2] \end{aligned}$$

for $0<\tau _n < t$, and $P(E_r,\tau _n\leftarrow e,0)=0$ otherwise.

The net probability of an outcome $\tau _n$ is

20 $$\begin{aligned} P(\tau _n\leftarrow e,0)= \int dE_r{\rho }(E_r) P(E_r,\tau _n\leftarrow e,0) \approx \frac{ \Gamma }{2\omega \sqrt{nN}}\exp [-\Gamma \tau _n] \end{aligned}$$

and replacing $\sum _n \rightarrow \int _0^t d\tau _n$ helps to verify that the overall decay rate is not affected by the presence of the clock, $P^{\text {decay}}_{\Delta t}(t)=\sum _n P(\tau _n\leftarrow e,0)=1-\exp (-\Gamma t)$. Finally, the spread of the energies of the emitted photons is no longer Lorentzian, but Gaussian,

21 $$\begin{aligned} P(E_r\leftarrow {e}, t\rightarrow \infty )= \sum _n P(E_r,\tau _n\leftarrow e,0) \approx \frac{\Delta t}{\sqrt{2\pi }}\exp [-(E_r-E_{e})^2\Delta t^2/2], \end{aligned}$$

and becomes broader as Alice’s accuracy improves, $\Delta t \rightarrow 0$. [Note that we cannot arrive at the Lorentzian distribution (8) simply by sending $\Delta t \rightarrow \infty$ in Eq. (21), since Eq. (20) was derived under assumption that the number of bosons in the right well is large].

### A clock which first waits and then runs

Alice can also consider a Markovian clock which starts running only after the transition has taken place and continues doing so until the time of observation *t*. (It will be clear shortly why this case is of interest). Replacing in Eq. (13) projector $\hat{\pi }_e$ by $1-\hat{\pi }_e=\int _{-\infty }^{\infty } dE_r |E_r {\rangle }{\langle }E_r |$, $\tau$ with $t-\tau$, and acting as before yields [see the “Methods” section (Coupling the clock to a quantum system)]

22 $$\begin{aligned} A^{\text {a+ph+clock}}_{\text {Bose}}(e,n\leftarrow e,0) = \exp [-i E_et-\Gamma t /2] \delta _{n0},\quad \quad \quad \quad \quad \quad \quad \quad \quad \quad \nonumber \\ A^{\text {a+ph+clock}}_{\text {Bose}}(E_r,n\leftarrow e,0) \approx \frac{(-i)^{n+1}\Omega }{[2\pi n]^{1/4}}\times \quad \quad \quad \quad \quad \quad \quad \quad \quad \quad \quad \quad \quad \quad \nonumber \\ \int _0^t \exp \left[ -\frac{(t_n-\tau )^2}{\Delta t^2}\right] \exp \{-iE_r\tau -i(E_e-i\Gamma /2)(t-\tau )\}d\tau ,\quad \quad \quad \quad \quad \end{aligned}$$

where $\delta _{n0}$ is the Kronecker delta. Now the number of the bosons in the right well is determined by the time which has elapsed since the moment of emission, and we can attend to the cat which dies as a result of the atom’s decay.

### Exploding powder kegs and poisoned cats

It is difficult to resist the temptation to relate the present discussion to the famous Schrödinger’s Cat problem. In 1935 Einstein and Schrödinger discussed a hypothetical case in which explosion of a powered keg was caused by a photon emitted by a decaying atom. In^6^ Schrödinger dramatised the narrative further by replacing the unstable powder by a now famous live cat, which dies in the event. The perceived contradiction was due to the fact that, prior to the final observation of the cat’s state, the wave function of the joint system was deemed to be a superposition of the states $|\text {atom: excited}{\rangle }\otimes |\text {cat: alive}{\rangle }$ and $|\text {atom: decayed}{\rangle }\otimes |\text {cat: dead}{\rangle }$. With wave function believed to reflect on the actual condition of a system, this left a big question mark over the cat’s situation prior to be found either dead or alive. The same contradiction was observed in the powder keg example, where, again, macroscopically distinguishable states $|\text {unexploded}{\rangle }$ and $|\text {exploded}{\rangle }$ were forced into superposition through entanglement with the atom.

It is worth revisiting the situation by replacing the cat (the keg) with the (nearly) classical clock of Section “A clock which first waits and then runs”. So far, the cat paradox did not arise because we only required a matrix element of a unitary operator $\hat{U}^{\text {a+ph+clock}}(t) =\exp (-i \hat{H}^{\text {a+ph+clock}}t)$ between the states $|E_r{\rangle }\otimes |n{\rangle }$ and $|e{\rangle }\otimes |0{\rangle }$ in the Hilbert space of the composite ${\text {a+ph+clock}}$. The question, we recall, was “is there a photon, and how many bosons are there in the right well at *t*?” Although there appears to be no need for it, one can create a kind of “cat” problem by looking at the ket

23 $$\begin{aligned} \hat{U}^{\text {a+ph+clock}}(t)|e,0{\rangle }= \exp [-i E_et-\Gamma /2t] |\Phi ^{\text {clock}}_{\text {Bose}}(0){\rangle }\otimes |e{\rangle }+ \nonumber \\ \sum _r \int _0^t d\tau ' A^{\text {a+ph}}(E_r\leftarrow e,t|t-\tau ') |\Phi ^{\text {clock}}_{\text {Bose}}(\tau ){\rangle }\otimes |E_r{\rangle }\end{aligned}$$

and object the appearance of a superposition of distinguishable macroscopic states in r.h.s. of Eq. (23). Indeed, for an accurate clock, i.e. $\Delta t \rightarrow 0$ ($N>>1$), the clock’s states in the r.h.s. of Eq. (23) are practically orthogonal [cf. Eq. (12)], ${\langle }\Phi ^{\text {clock}}_{\text {Bose}}(\tau ')|\Phi ^{\text {clock}}_{\text {Bose}}(\tau ){\rangle }\sim \exp \left[ -{(\tau -\tau ')^2}/{\Delta t^2}\right] \xrightarrow [\Delta t \rightarrow 0]{} 0$. Alternatively, one can avoid the paradox of the cat being both dead and alive by considering the superposition to be a transient artefact of the calculation, needed only to establish the likelihood of finding *n* escaped bosons, and having no further significance.

**Figure 3:**
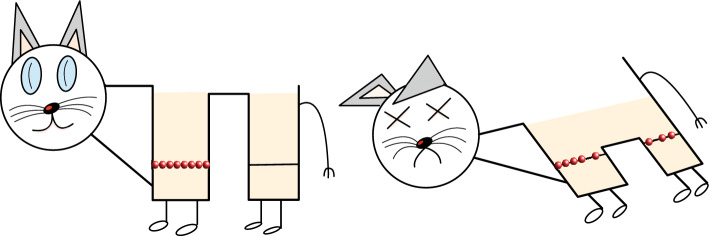
An artists’s impression of a primitive cat (**a**) alive and well, and (**b**) sadly, dead for some time. Any resemblance to real cats, living or dead, is purely coincidental.

The analogy can be taken further. Neither the cat’s demise, nor an explosion are purely instantaneous events. By looking at the deterioration of the cat’s body (we leave outside the question of what it means to be alive) one can tell how long ago it stopped functioning. By looking at how much of the powder has been burnt, or how much dust thrown up in the air has settled, it is possible to deduce the moment when explosion started. Remarkably, the waiting clock of Section “A clock which first waits and then runs” keeps a similar record, only in a more direct way (Fig. 3).

Alice may find no bosons in the right well (cat is alive), or a certain number of them (a particular stage of decay of the dead cat’s body). The more accurately Alice is able to deduce the “moment of death”, the broader will be the energy distribution of the photon whose emission has killed the cat [cf. Eq. (21)]. A valid analogy could be a very long fuse, whose burnt length (number of bosons in the right well) would let one deduce the moment when it was set on fire.

### Beyond the wide band approximation

Next we revisit a more general (non-Markovian) case of Section “Results and discussion” [cf. Eq. (2)], where the product $|\Omega (E_r)|^2{\rho }(E_r)$ may depend on the photon’s energy, $\int _{-\infty }^\infty dE |\Omega (E_r)|^2{\rho }(E_r)$ is finite, and the transition occurs via a single jump. Only a small proportion of all atoms will be found decayed by the time *t*, but Alice may still want to know when this unlikely transition did occur. A simple calculation [see the “Methods” section (Timing the first order transition in a non-Markovian case)] shows that the probability of the clock’s reading $\tau _n$ for a system ending in a state $|E_r{\rangle }$, is still given by an expression similar to Eq. (19),

24 $$\begin{aligned} P(E_r,n\leftarrow e,0,t)\approx \frac{\pi \Omega ^2(E_r) \Delta t^2}{[2\pi n]^{1/2}} \exp [-(E_r-E_e)^2\Delta t^2/2], \end{aligned}$$

so that measuring the moment of emission to an accuracy $\Delta t$ broadens the range of the photon’s energies, which grows as $1/\Delta t$ owing to the Gaussian in r.h.s. of Eq. (24). Therefore, it is the availability of the final system’s states that restricts the decay rate, and is responsible for the Zeno effect already mentioned in Section “Results and discussion”. Indeed, acting as before (cf. Section “Methods”), for the probability to decay by the time *t* we find

25 $$\begin{aligned}{} & {} P^{\text {decay}}_{\Delta t}(t) =\sum _n\int dE_r{\rho }(E_r)P(E_r,n\leftarrow e,0,t)= \Gamma _{\Delta t}\times t, \end{aligned}$$

26 $$\begin{aligned}{} & {} \Gamma _{\Delta t} \equiv \sqrt{2\pi } \Delta t \int dE_r{\rho }(E_r)\Omega ^2(E_r) \exp [-(E_r-E_{e})^2\Delta t^2/2]. \end{aligned}$$

In the Markovian wide band limit $\Gamma _{\Delta t}$ in Eq. (26) does reduce to Fermi’s golden rule^11^, $\Gamma _{\Delta t}=\Gamma _{\text {Fermi}}=2\pi \Omega ^2 {\rho }$. But if the integration of an ever broader Gaussian is restricted to a finite range, the factor of $\Delta t$ in Eq. (26) is no longer cancelled, and the decay rate eventually decreases as the measurement becomes more accurate. For example, consider a special case of an energy band of a width $\Delta E_r=E_{\text {max}}-E_{\text {min}}$, wherein ${\rho }(E_r)\Omega ^2(E_r)=const$. Comparing the decay rates prescribed by Eq. (26) and by Fermi’s rule, we have

27 $$\begin{aligned} \Gamma _{\Delta t}/\Gamma _{\text {Fermi}}\xrightarrow [\Delta t \rightarrow 0]{}\frac{\Delta E_r\Delta t}{\sqrt{2\pi }}. \end{aligned}$$

**Figure 4:**
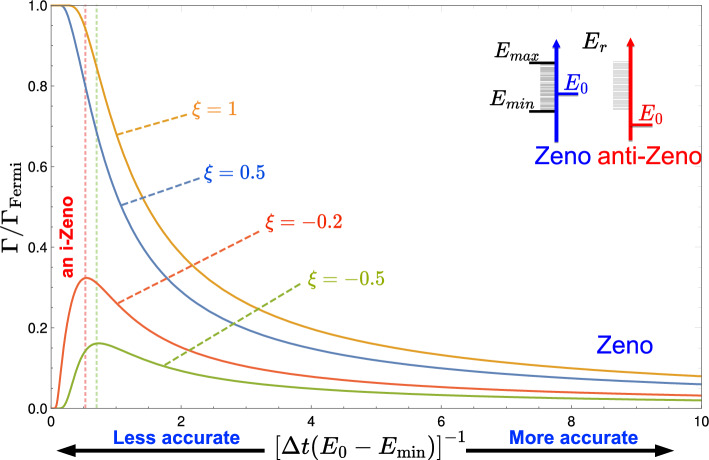
The rate of decay into a finite-sized band $E_{\text {min}}\le E_r\le E_{\text {max}}$ as a function of the clock’s accuracy $\Delta t$ [$\xi = 2(E_e-E_{\text {min}})/\Delta E_r$]. For $E_{\text {min}}< E_e< E_{\text {max}}$ better accuracy means a smaller decay rate (Zeno effect); for $E_e < E_{\text {min}}$ there is an initial increase in the value of $\Gamma$ (anti-Zeno effect). Both possibilities are illustrated in the inset. The anti-Zeno effect also occurs in the case $E_e >E_{\text {max}}$, not shown here.

The accuracy with which the moment of emission can be determined without significantly altering the decay rate is, ultimately, limited by the width of the energy range, available to the emitted photon. What happens for not too small values of $\Delta t$ depends, however, on whether the excited atom’s energy lies within the allowed range, as explained in Fig. 4. If $E_e <E_{\text {min}}$ or $E_e >E_{\text {max}}$ unobserved atom cannot decay, and the decay rate first increases as $\Delta t$ becomes smaller, leading to a kind of “anti-Zeno” effect^18^. It eventually begins to fall off in agreement with Eq. (27), when the exponential in Eq. (24) can be approximated by unity.

With all this in mind, we can revisit the analysis of^5^, where the *duration* of a jump was estimated in the following manner. Every $\delta t$ seconds one checks whether the atom continues in its excited state. The jump time, $\tau _{\text {J}}$, is then taken to be the $\delta t$ for which the checks begin to affect the atom’s decay rate. For $\tau _{\text {J}}$,^5^ finds

28 $$\begin{aligned} \tau _{\text {J}}\approx \Gamma _{\text {Fermi}}\tau _{\text {z}}^2, \end{aligned}$$

where $\tau _{\text {z}}\equiv \left[ {\langle }e|\hat{H}^2_{\text {a+ph}}|e{\rangle }-{\langle }e|\hat{H}_{\text {a+ph}}|e{\rangle }^2\right] ^{-1/2}$ is the “Zeno time”. In the regime studied in^5^ $\delta t$ is short enough for the transition to occur via a single jump. One can, therefore, equally interpret $\tau _{\text {J}}$ as the *uncertainty* in the moment at which an instantaneous transition takes place. In the case we have studied here, Alice’s clock begins to affect the decay rate when its error is of order of the inverse band width, $\Delta t \sim 1/\Delta E_r$ [cf. Eq. (27)]. It is easy to check (see the “Methods” section [The “jump time” in Eq. (28))] that Eq. (28) yields a similar result, $\tau _{\text {J}} \sim 1/\Delta E_r$.

### Feynman’s “only mystery of quantum mechanics”

All this leaves one with a question: “what can be said about the moment of emission if it has not been timed by a cat, gunpowder, or a clock?” Very little, according to Feynman^7,19^. In a double slit experiment a particle can reach a point on the screen by passing through the holes, with the probability amplitudes $A_1$ and $A_2$, respectively. The probability of arriving at the screen with both slits open is $|A_1+A_2|^2$, while with only the first one open it is $|A_1|^2$. With no restriction on the signs of the amplitudes, it is possible to have (e.g., near a dark fringe) $|A_1+A_2|^2<|A_1|^2$, so that eliminating one of the routes increases the number of arriving particles. For this reason, it is not possible to assume that a setting of the particle’s internal machinery (or any other hidden variable) predetermines the hole to be chosen by each particle on its way to the screen. The mathematics cannot be simpler, and one must conclude that “... when you have no apparatus to determine through which hole the thing goes, then you cannot say that it either goes through one hole or the other”. This is an illustration of the Uncertainty Principle^7^ which states that one cannot determine which of the alternatives has been taken without destroying interference between them.

**Figure 5:**
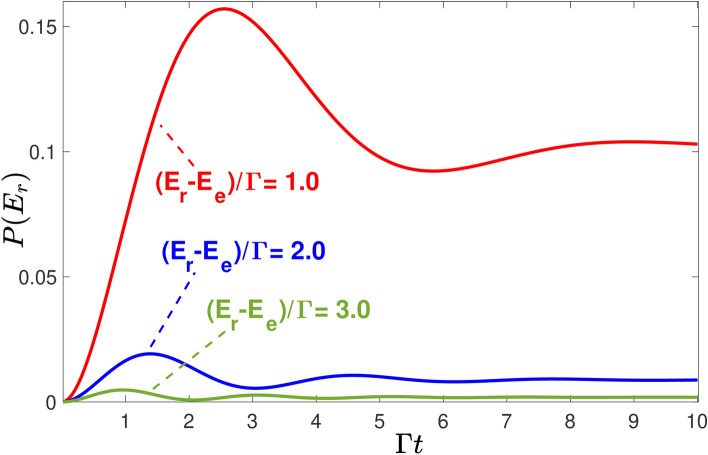
Probability of finding the photon in a unit interval around energy $E_r$ in a single measurement made at time *t*. Note that similar results were observed in^20^.

The same principle, applied to the case of a decaying atom, states that with no apparatus to determine the moment of decay, one cannot say that the atom emits a photon with an energy between $E_r$ and $E_r+dE_r$ at one moment or the other. Indeed, if each atom were predestined to decay at a given time, the number of decayed atoms could only *increase* or stay the same as the time span available for the atom’s decay becomes longer. However, the corresponding probability is given by $W(E_r,E_r+dE_r)=P(E_r) dE_r$, and $P(E_r)=\rho |\int _0^t A^{\text {a+ph}}(E_r\leftarrow e,t|\tau ) d\tau |^2$, shown in Fig. 5, can *decrease* with *t*. (Note that the probability in Fig. 5 is that of a single measurement made at different times. If the decayed atoms are counted twice, the number measured at a later time is, of course, always greater.) The decrease cannot be blamed on the re-absorption of the photon, impossible in the Markovian model [cf. Eq. (6)]. Neither can it be explained by the change in the emitted photon’s energy [cf. Eq. (7)].

This seems even stranger than the double slit case. One could imagine the routes passing through different holes merged, like two confluent rivers, where it is impossible to say on which of the two a boat is. Merging time intervals may be even more difficult to fathom, but to conclude that an unobserved transition has occurred at a particular moment would lead to “an error in prediction”^19^, as was discussed above. This is, according to Feynman^7^, the only mystery of quantum mechanics, which defies “any deeper explanation”.

## Conclusions

The story of the Schrödinger’s cat, whose death is caused by the decay of an excited atom, is one of the best known illustration of a problem which one expects to arise when the classical world meets its quantum counterpart^6^. A classical system, believed to have an unbroken continuous history, appears to loose this property if forced to interact with a quantum object, for which no continuous description is thought to be available^21^. To bridge the gap between the classical and quantum views we design a nearly classical macroscopic clock, capable of timing the moment of decay to a good, yet finite accuracy. The complete narrative is as follows.

An atom, prepared in its excited state is found decayed at time *t*, after having emitted a photon with energy $E_r$. The instant of emission is unknown, and to determine it the experimenter needs a device which would measure it. One suitable choice is a clock consisting of large number *N* of noninteracting bosonic atoms, initially trapped in the left well of a double well potential. Finding that *n* bosons have made the transition to the right well, one can estimate the elapsed time *t* as $t_n \approx \omega ^{-1}\sqrt{n/N}$, with an error $\Delta t \approx \omega ^{-1}/\sqrt{N}$. With the transition amplitude $\omega$ small, and the number of bosons large, $N>>1$ the clock is a source of irreversible current flowing from left to right. With many bosons in the right well, $N>>n>>1$, the clock is seen to acquire an important classical property. Its wave function becomes localised, and one is able to measure time to a good accuracy without significantly perturbing the clock’s evolution (for more details see also^14^).

The clock can be arranged to run until the moment of emission, which would yield a good estimate of the time of emission provided $\Delta t/t_n<< 1$, except in the unlikely case of the decay occurring almost immediately. The effect of the measurement on the atom’s decay depends on the range of energies $\Delta E_r$, available to the emitted photon. In the wide band limit, $\Delta E_r\Delta t>>1$ the decay rate $\Gamma$ remains the same, and destruction of interference between the moments of emission leads only to broadening of the photon’s energy spectrum, whose shape is no longer Lorentzian, but Gaussian, with a width $\sim 1/\Delta t$. Having obtained a result $t_n$, and knowing that more measurements could have been added both before and after $t=t_n$ (almost) without altering the clock’s evolution, the experimenter has a complete history of what has happened. The atom remained in its excited state until $t_n-\Delta t \lesssim t_n+\Delta t$, and then continued in the ground state until the time when the clock is read. Note that essential for recovering such a continuous description is the classical property of the macroscopic clock reached in the limit $N>>1$.

The Zeno effect sets in when the inverse clock’s accuracy become comparable to the range of available photon’s energies, $\Delta E_r\Delta t\lesssim 1$. Now the notion of the moment of decay is meaningful only in the weak coupling limit, $\Gamma _{\text {Fermi}} t<<1$ [cf. Eq. (2)]. In the “narrow band” limit, $\Delta E_r\Delta t<< 1$, the decay rate is proportional to $\Delta t$, and the unlikely atomic decay is further suppressed as $\Delta t \rightarrow 0$.

The clock set up to run after the decay has occurred, helps provide an additional insight into the fate of the Schrödinger’s feline^6^. Now one knows that there were no bosons in the right well until $t_n$ (within an error margin $\Delta t$), after which their number there was steadily growing. One can leave the question of what it means to be alive outside the scope of quantum theory, and concentrate instead on the deterioration of the cat’s macroscopic physical body. The waiting clock is a blueprint for a very primitive “cat”, said to be alive if there are no bosons in the right well, $n=0$, and dead in some stage of decay with $n>>1$. If the analogy holds, a real cat’s physical frame should be characterised by a quantum uncertainty $\Delta t_{\text {cat}}$, which limits the ability of an experienced forensic scientist to determine the time of death by studying the cat’s remains. The cat’s fate depends, therefore, on the details of the atom’s decay. In the wide band model, the probability of survival up to time *t* decreases as $\exp (-\Gamma t)$, regardless of the $\Delta t_{\text {cat}}$. However, in the finite band case, a cat whose body would allow to determine the moment of death with greater precision should have a better chance to survive its ordeal.

## Methods

### Derivation of Eq. (11)

The normal approximation to $A^{\text {clock}}_{\text {Bose}} (n\leftarrow 0,t)= (-i)^n\sqrt{C^N_n p^n(1-p)^{N-n}}$ reads ($p(t)=\omega ^2t^2<<1$)

29 $$\begin{aligned} A^{\text {clock}}_{\text {Bose}}(n\leftarrow 0,t)\approx (-i)^n(2\pi Np)^{-1/4}\exp [-(n-Np)^2/4Np]. \end{aligned}$$

In new variables $t_n\equiv \omega ^{-1}\sqrt{n/N}$ and $\Delta t \equiv \omega ^{-1}N^{-1/2}$ we have

30 $$\begin{aligned} A^{\text {clock}}_{\text {Bose}}(n\leftarrow 0,t)\approx (-i)^n (2\pi N \omega ^2t^2)^{-1/4}\exp [-(t^2_n-t^2)^2/4t^2\Delta t^2]. \end{aligned}$$

As $N\rightarrow \infty$, $\Delta t \rightarrow 0$, and the exponential is sharply peaked around $t_n \sim t$, or $n\sim N \omega ^2 t^2$, and the amplitude can be approximated by

31 $$\begin{aligned} A^{\text {clock}}_{\text {Bose}}(n\leftarrow 0,t)\approx (-i)^n(2\pi n)^{-1/4}\exp [-(t_n-t)^2/\Delta t^2], \end{aligned}$$

which is Eq. (11).

### A macroscopic clock

Consider *N* non-interacting bosons, each occupying the same state $|\phi {\rangle }$, $|\Phi {\rangle }=\prod _{i=1}^N |\phi {\rangle }_i$. Expanding the $|\phi {\rangle }$s in an orthonormal basis $|j{\rangle }$, $j=1,2$, $|\phi {\rangle }= \alpha |1{\rangle }+\beta |2{\rangle }$ yields

32 $$\begin{aligned} |\Phi {\rangle }= \sum _{n_1=0}^N B_{n_1}|n_1,N{\rangle },\quad B_{n_1}=\sqrt{C^N_{n_1}} \alpha ^{n_1}\beta ^{N-n_1}, \end{aligned}$$

where $C^N_{n_1}$ is the binomial coefficient, and $|n_1,N{\rangle }$ describes a state with $n_1$ particles populating the state $|1{\rangle }$. Suppose one wants to measure the number of particles in the state $|1{\rangle }$, $\hat{N}_{1} =\sum _{i=1}^N |1{\rangle }_i{\langle }1|_i$, using a Gaussian von Neumann pointer, whose initial state is $G(f)=C \exp [-f^2/\Delta f^2]$. After the measurement for the entangled state of the pointer and the bosons, $|\Phi {\rangle }$, one finds

33 $$\begin{aligned} {\langle }f|\Phi {\rangle }= \sum _{n_1=0}^N B_{n_1}G(f-n_1)|n_1,N{\rangle }. \end{aligned}$$

The distribution of the pointer’s readings *f* and the mixed state of the bosons $\hat{\rho }$ are, therefore, given by

34 $$\begin{aligned} w(f)= \sum _{n_1=0}^N |B_{n_1}|^2G^2(f-n_1), \end{aligned}$$

and

35 $$\begin{aligned} \hat{\rho }=\sum _{n_1, n_1'=0}^N B^*_{n_1'}B_{n_1}I_{n_1'n_1}|n_1,N{\rangle }{\langle }n_1',N|, \quad I_{n_1'n_1}\equiv \int G(f-n_1')G(f-n_1)df. \end{aligned}$$

With $N,|\alpha |^2N>>1$ the readings lie near the mean value $\overline{n}_1=|\alpha |^2N$. Using the normal approximation for the binomial distribution $|B_{n_1}|^2$, and replacing the sum by an integral yields

36 $$\begin{aligned} w(f)\approx \frac{C^2}{\sigma \sqrt{2\pi }} \int \exp \left[ -\frac{(n_1-\overline{n}_1)^2}{2\sigma ^2}-\frac{2(f-n_1)^2}{\Delta f ^2}\right] dn_1\sim \exp \left[ -\frac{2(f-\overline{n}_1)^2}{\Delta f^2+4\sigma ^2}\right] , \end{aligned}$$

where $\sigma =\sqrt{N |\alpha |^2(1- |\alpha |^2)}$. For a large *N* it is possible to choose $\sigma<< \Delta f<< \overline{n}_1$. This yields a good measurement, $w(f)\sim \exp \left[ -2(f-\overline{n}_1)^2/\Delta f^2\right]$, with a relative error $\sim \Delta f /\overline{n}_1<<1$. What is more, since the non-zero $B_{n_1}$s lie within a range $\sim \sigma$ around $\overline{n}_1$, all relevant factors $I_{n_1'n_1}$ in Eq. (35) can be replaced by unity. Thus, the bosons’ state is almost unperturbed by a good, yet weakly perturbing measurement, and is ready for the next observation. Since the choice of the basis $|j{\rangle }$ is arbitrary, one can say that, for a large system, different collective (macroscopic) variables acquire well-defined “classical” values even when the corresponding one-particle projectors $\hat{n}_1=|1{\rangle }{\langle }1|$ and $\hat{n}_{1'}=|1'{\rangle }{\langle }1'|$ do not commute. By the same token, the progress of a large system can be monitored by consecutive measurements of the same macroscopic quantity without seriously affecting its evolution. This is “classicality by numbers”^14^.

### Coupling the clock to a quantum system

Consider an evolution operator for a system (s) coupled to a clock,

37 $$\begin{aligned} \hat{U}^{\text {s+clock}}(t)=\exp [-i(\hat{H}^{\text {s}} + \hat{\pi }\hat{H}^{\text {clock}})t], \end{aligned}$$

where $\hat{\pi }$ projects onto a sub-space *h* of the system’s Hilbert space. Since $\hat{H}^{\text {clock}}$ commutes with both $\hat{H}^{\text {s}}$ and $\hat{\pi }$ we can write ($\delta (x)$ is the Dirac delta, and $\lambda$ is a *c*-number)

38 $$\begin{aligned} \hat{U}^{\text {s+clock}}(t)=\int _{-\infty }^\infty d\lambda \delta (\lambda - \hat{H}^{\text {clock}}) \exp [-i(\hat{H}^{\text {s}} +\lambda \hat{\pi })t]. \end{aligned}$$

[It is also straightforward to verify that the r.h.s. of Eq. (38) satisfies the correct equation of motion, $i\frac{d}{dt}\hat{U}^{\text {s+clock}]}=\left( \hat{H}^{\text {s}} + \hat{\pi }\hat{H}^{\text {clock}}\right) \exp (-i\left( \hat{H}^{\text {s}} + \hat{\pi }\hat{H}^{\text {clock}}\right) t)$]. But $\delta (\lambda - \hat{H}^{\text {clock}})=(2\pi )^{-1}\int _{-\infty }^\infty d\tau \exp (i\lambda \tau - i\hat{H}^{\text {clock}}\tau )$, and we have

39 $$\begin{aligned} \hat{U}^{\text {s+clock}}(t)=\int _{-\infty }^\infty d\tau \hat{U}^{\text {s}}(t|\tau )\hat{U}^{\text {clock}}(\tau ), \end{aligned}$$

where $\hat{U}^{\text {s}}(t|\tau )=(2\pi )^{-1}\int _{-\infty }^\infty d\lambda \exp (i\lambda \tau ) \exp [-i(\hat{H}^{\text {s}} + \lambda \hat{\pi })t]$ evolves the system under an additional condition that it must spend $\tau$ seconds in the chosen sub-space, and $\hat{U}^{\text {clock}}(\tau )=\exp (-i \hat{H}^{\text {clock}}\tau )$ evolves the clock for precisely $\tau$ seconds.

If the clock is set to measure the duration spent by the system in the sub-space orthogonal to *h*, $\hat{\pi }$ is replaced by $1-\hat{\pi }$, and Eq. (39) becomes

40 $$\begin{aligned} \hat{U}^{\text {s+clock}}(t)=\int \hat{U}^{\text {s}}(t|\tau )\hat{U}^{\text {clock}}(t-\tau )d\tau = \int \hat{U}^{\text {s}}(t|t-\tau )\hat{U}^{\text {clock}}(\tau )d\tau \end{aligned}$$

with the clock running whenever the system is *not * in the subspace *h*. For a transition amplitude between states $|\psi ^{\text {s}}_i{\rangle }|\phi ^{\text {clock}}_i{\rangle }$ and $|\psi ^{\text {s}}_f{\rangle }|\phi ^{\text {clock}}_f{\rangle }$, $A^{\text {s+clock}}(\psi ^{\text {s}}_f,\phi ^{\text {clock}}_f\leftarrow \psi ^{\text {s}}_i,\phi ^{\text {clock}}_i,t)= {\langle }\psi ^{\text {s}}_f|{\langle }\phi ^{\text {clock}}_f|\exp (-i\hat{H}^{\text {s+clock}}t)|\psi ^{\text {s}}_i{\rangle }|\phi ^{\text {clock}}_i{\rangle }$ we have

41 $$\begin{aligned} A^{\text {s+clock}}(\psi ^{\text {s}}_f,\phi ^{\text {clock}}_f\leftarrow \psi ^{\text {s}}_i,\phi ^{\text {clock}}_i,t)=\int A^{\text {s}}(\psi ^{\text {s}}_f\leftarrow \psi ^{\text {s}}_i,t|\tau )A^{\text {clock}}(\phi ^{\text {clock}}_f\leftarrow \phi ^{\text {clock}}_i,\tau )d\tau , \end{aligned}$$

where $A^{\text {s}}(\psi ^{\text {s}}_f\leftarrow \psi ^{\text {s}}_i,t|\tau )$ is the amplitude of the system found in its final state while spending a duration $\tau$ in *h*, and $A^{\text {clock}}(\phi ^{\text {clock}}_f\leftarrow \phi ^{\text {clock}}_i,\tau )$ is that of the clock reaching $|\psi ^{\text {s}}_i{\rangle }$ after $\tau$ seconds. For a clock measuring the duration spent in the part of the Hilbert space, orthogonal to *h*, $\tau$ should be replaced by $t-\tau$ as it has been done in Eq. (40).

### Timing the transition in the Markovian case

Now the system including the atom and a photon (if any) is described by the Hamiltonian (1), $\hat{\pi }_e \equiv |e{\rangle }{\langle }e|$ projects onto the atom’s excited state (no photons). Thus introducing $\lambda \hat{\pi }_e$ to the Hamiltonian simply adds $\lambda$ to the energy of the excited state $E_{e}\rightarrow {E_{e}} +\lambda$. We may evaluate the amplitudes for the modified Hamiltonian, $\hat{H}+\lambda \hat{\pi }_e$ and then perform the Fourier transform. We have

42 $$\begin{aligned} A^{\text {a+ph}}(e\leftarrow e,t{}|\tau ) &=(2\pi )^{-1}\int _{-\infty }^\infty d\lambda \exp [i\lambda \tau -i({E_{e}}+\lambda ) t -\Gamma t/2]\nonumber \\ &=\exp (-iE_{e}t -\Gamma t/2)\delta (\tau -t).\quad \quad \quad \quad \quad \quad \quad \quad \quad \quad \quad \quad \end{aligned}$$

Similarly, we find

43 $$\begin{aligned} A^{\text {a+ph}}(E_r\leftarrow e,t{}|\tau ) = -i\Omega \exp (-iE_rt) \int _0^t dt'\exp [-i(E_{e}-E_r) t' -\Gamma t'/2]\delta (\tau -t')\nonumber \\ ={\left\{ \begin{array}{ll} -i\Omega \exp [-iE_r(t-\tau )] \exp [{-iE_{e}\tau -\Gamma \tau /2}]\quad \text{ for }\quad 0\le \tau \le t\\ 0\quad \text {otherwise} \end{array}\right. }, \end{aligned}$$

which is the second of Eq. (16). The remaining amplitudes are

44 $$\begin{aligned} A^{\text {a+ph}}(e\leftarrow E_r,t{}|\tau ) =0 \end{aligned}$$

and

45 $$\begin{aligned} A^{\text {a+ph}}(E_r'\leftarrow E_r,t{}|\tau ) =\exp (-iE_rt)\delta (E_r-E_r')\delta (\tau ). \end{aligned}$$

### Timing the first-order transition in a non-Markovian case

In the general non-Markovian case, to calculate the required amplitude we expand, to the first order in $\hat{V}$, a transition amplitude

46 $$\begin{aligned} {\langle }E_r|\exp [-i(\hat{H}+\lambda \hat{\pi }_e)t]|e{\rangle }\approx -i\sum _{r'} \Omega (E_{r'}) \int _0^t dt'\times \quad \quad \nonumber \\ {\langle }E_r|\exp [-i(\hat{H}_0+\lambda \hat{\pi }_e)(t-t')]E_{r'}{\rangle }{\langle }e|\exp [-i(\hat{H}_0+\lambda \hat{\pi }_e)t']|e{\rangle }. \end{aligned}$$

The integrand reduces to [recall that adding $\lambda \hat{\pi }_e$ changes $E_{e}$ into $E_e+\lambda$ in Eq. (1)]

47 $$\begin{aligned} \exp [-iE_r(t-t')]\delta _{r'r} \exp [-i(E_e+\lambda )t'], \end{aligned}$$

and performing the Fourier transform with respect to $\lambda$ yields

48 $$\begin{aligned} A^{\text {a+ph}}(E_r\leftarrow e,t|\tau ) ={\left\{ \begin{array}{ll} -i\Omega (E_r)\exp [-iE_r(t-\tau )] \exp [-iE_{e}\tau ]\quad \text{ for }\quad 0\le \tau \le t\\ 0\quad \text {otherwise} \end{array}\right. }.\quad \end{aligned}$$

Using Eqs. (11), (14) and (48) we find

49 $$\begin{aligned} A^{\text {a+ph+clock}}_{\text {Bose}}(E_r,n\leftarrow e,0)=const\times \int _0^t \exp [-(\tau -\tau _n)^2/\Delta t^2+i(E_r-E_e)\tau ]d\tau . \end{aligned}$$

For $\Delta t \rightarrow 0$ the limits of integration can be extended to $\pm \infty$. Evaluating the Gaussian integral, and taking the absolute square then yields

50 $$\begin{aligned} P(E_r,n\leftarrow e,0,t)\approx \frac{\pi \Omega ^2(E_r) \Delta t^2}{[2\pi n]^{1/2}} \exp [-(E_r-E_e)^2\Delta t^2/2]. \end{aligned}$$

Replacing ($n_{\text {max}}=\omega ^2t^2N$) the sum $\sum _{n=0}^{n_{\text {max}}}n^{-1/2}$ by an integral $\int _0^{n_{\text {max}}}n^{-1/2}dn= 2\sqrt{n_{\text {max}}}=2 t/\Delta t$ we obtain the energy distribution of the photons in the presence of a clock

51 $$\begin{aligned} P(E_r\leftarrow 0,t)= \sum _{n=0}^{n_{\text {max}}}P(E_r,n\leftarrow e,0,t)\approx {\sqrt{2\pi } \Omega ^2(E_r)\rho (E_r) \Delta t} \exp [-(E_r-E_e)^2\Delta t^2/2]\times t. \end{aligned}$$

### The “jump time” in Eq. (28)

Let the decay occur into a finite energy range $\Delta E_r =E_{\text {max}}-E_{\text {min}}$ around $E_{e}$, and assume that ${\rho }(E_r)\Omega ^2(E_r)= const$ inside the range, and vanishes outside it. Using Hamiltonian (1), for the Zeno time we have

52 $$\begin{aligned} \tau _{\text {z}}^2\equiv \left[ {\langle }e|\hat{H}^2|e{\rangle }-{\langle }e|\hat{H}|e{\rangle }^2\right] ^{-1} = [{\rho }\Omega ^2 \Delta E_r]^{-1}. \end{aligned}$$

Recalling that $\Gamma _{\text {Fermi}}=2\pi {\rho }(E_{e})|{\langle }E_r=E_e |\hat{H}|E_e{\rangle }|^2=2\pi {\rho }\Omega ^2$ shows that Eq. (28) reduces to

53 $$\begin{aligned} \tau _{\text {J}} \approx 2\pi /\Delta E_r \sim 1/\Delta E_r. \end{aligned}$$

## Data Availability

The datasets used and/or analysed during the current study available from the corresponding author on reasonable request.
